# Women Empowerment or Feminism: Facts and Myths about Feminization of Medical Education

**DOI:** 10.12669/pjms.36.3.2396

**Published:** 2020

**Authors:** Lubna Ansari Baig

**Affiliations:** Dean APPNA Institute of Public Health Jinnah Sindh Medical University Karachi - Pakistan

From medieval times and through the middle of 20^th^ century women were extinct and in some cases excluded from medical schools. By the end of 19^th^ century this extinction came to an end and women medical colleges were built in some parts of the world. By the end of the 20^th^ century the scenario changed and more females entered and graduated from medical schools.^1^ This phenomena of more females entering medical profession compared to males is referred to as “the Feminization of medicine”.^1^-^3^ It is imperative to look at this term carefully. As per the Webster dictionary it is acquisition of female characteristics by males or females.^4^ Feminization of medical education is referred to as induction of females in Medicine.^1^ Let us look at the long struggle of women all over the world and Pakistan in getting their needed and due place in the health system.

Before the 1400 AD license was not required to practice medicine in Europe and women were providing care through remedies or nursing or being herbalists.^5^ After 1400 as women were not allowed to get admission in medical schools in the West, they obviously did not get a license and were not allowed to practice medicine.^5^ From that time onwards the Medical Profession became male dominated and women became extinct as medical practitioners. This then prompted wealthy women and some countries with means to start female only medical schools and some with special seats for females.

The first female only medical school was founded in Boston in 1848 (New England Female Medical College) followed by Woman’s Medical College of Pennsylvania (founded 1850) in USA. In Europe Ms. Sophia Jex-Blake founded two medical schools in United Kingdom; London School of Medicine for Women (founded 1874) and Edinburgh School of Medicine for Women (founded 1886).^5^ In the Far East Asia Tokyo Women’s Medical University was founded in 1900 by Yoshioka Yayoi and Hackett Medical College for Women, Guangzhou, China, founded in 1902 by Presbyterian Church.^1^,^5^ This then started a wave of more females entering into Medical Schools and getting license to practice in Europe and USA from the end of the 19^th^ century to the beginning of 20^th^ century.^6^ The women in United Kingdom now make almost 50% of the health workforce, however other disciplines like architecture and engineering are still male dominated.^6^

For managing this discrepancy in Punjab, Fatima Jinnah Medical College was established in 1948 by the struggle of Begum Rana Liaqut Ali and Mohtarma Fatima Jinnah to promote females in medical education. Later Shaheed Zulfiqar Ali Bhutto also established in 1973 a female medical college in Nawabshah, Sindh to meet the need for female medical practitioners. Before 1990 the admissions to medical colleges in Pakistan were based on quota whereby female seats were 30-40% or even less in some medical colleges. The females entering medical colleges still made less than 30% of the total medical students all over Pakistan. Girls started struggle to get seats in medical colleges on open Merit. In the famous Shirin Munir case by a Supreme Court order in 1990, this was changed to open merit admission all over Pakistan.^7^ This was appreciated all over and lead to higher number of females in medical and dental colleges of Pakistan. At this time the ratio of females to males in medical colleges of Pakistan is 3:1 or even 4:1 in some colleges.

This started a discussion on why fewer males are entering medical colleges and it was reported that they don’t find it financially attractive.^8^ It was also reported that girls are excelling in studies (High Schools & Medical College) and way ahead of boys.^9^ Ironically at the same time multiple papers reported that *“Medical degree is a hot ticket in the marriage market”.^9^* Daily Dawn one of the most popular news paper of Pakistan reported that Female doctors are popular in the marriage market as mothers-in-law prefer a *Doctor Daughter in Law (Bahu)*. It is a sign of pride and stature to have lady doctor as a trophy wife.^8^

Despite the feminization of medical education in the recent era women are not acquiring leading positions in academic medicine in the West as well.^10^ Mangan has referred to careers of female physicians as a “leaking pipeline” because of higher number of female entering the profession but disproportionately very low percentage of female physicians arriving at leading positions.^11^ Needless to say the career pathway in medical profession for men and women are significantly different. Males are predominantly in higher positions and women drop out at higher number. One possible explanation could be their shying away from academic medicine and other more challenging medical leadership positions.^12^

In Pakistan around 85,000 female doctors are not working and marriage has been reported as the major factor for this drop out.^13^ Multiple other reasons of this drop out have also been reported including: restriction by their husbands or in-laws, child bearing and rearing, harassment, inequality in promotions and salary structure, lack of flexible training schedules, supervisors preferential treatment, work schedules, etc.^14^ It has been reported that women physicians face discrimination from patients, nurses, male peers and supervising surgeons in countries that have similar cultural norms.^15^

Ensuring the strength of the physician workforce is essential to optimizing patient care. Challenges that undermine the profession include inequities in advancement, high levels of burnout, reduced career duration, and elevated risk for mental health problems, including suicide.^16^ Although starting from the ancient era to the present world, the role of Women in medicine and healing is proactive and evident throughout the history, albeit in different forms and with various associated conflicts along the way.^17^ It is evident that the participation of the young women has been on rise in education specially in the field of medicine in recent years and the strength of women enrolled in medical schools and residency programs support this evidence greatly but unfortunately a large number of them do not join the workforce for various reasons.^18^

While some attrition is expected in medicine, rates of attrition among women remain higher than men. This poses a problem for both the individuals who leave and the programs that have trained them, as significant time and resources have already been invested. More broadly, attrition affects the public due to the shortage of physicians in Pakistan. Furthermore, as a Muslim country Pakistan needs more practicing women physicians especially for conservative rural areas where there is a need to manage female patients.^19^

The time has come to dispel the myth that women enter medical schools for becoming trophy wives. The number of women doctors will increase as they are excelling in Education and gaining seats in medical and dental colleges on merit and not on quota. Women are the backbone of the healthcare system especially for countries like Pakistan where it is easy to communicate with same gender health care providers. Expectations of the society that women doctors should continue to work should match with the realization of her pivotal role in nurturing and rearing a family. Together we need to work towards building a strong social, educational and work support system that will help women and their families to achieve their dreams and aspirations and support the health care system.

To better integrate women in health workforce the first step is to revise the admission criteria (for both men and women) and in addition to test of Cognition, traits like: aptitude for medicine, future goals, family support system, ability to sustain pressure, communication and related skills should also be included. The educational and working environment should be made more women-friendly by training schedules that have flexible hours so that child bearing and rearing can be managed. Women should have an option for staggered entry and time to complete post graduation may be extended in special circumstances. There should be availability of child care at the training sites and places of work to ensure women’s professional development and provide equal opportunity for growth and development. Finally we need to look at it as women empowerment and not feminization of medical education. Women should be provided equal opportunity for training and job placements.
